# Applications and Perspectives of Cascade Reactions in Bacterial Infection Control

**DOI:** 10.3389/fchem.2019.00861

**Published:** 2020-01-08

**Authors:** Yuanfeng Li, Guang Yang, Yijin Ren, Linqi Shi, Rujiang Ma, Henny C. van der Mei, Henk J. Busscher

**Affiliations:** ^1^State Key Laboratory of Medicinal Chemical Biology, Key Laboratory of Functional Polymer Materials, Ministry of Education, Institute of Polymer Chemistry, College of Chemistry, Nankai University, Tianjin, China; ^2^Department of Biomedical Engineering, University of Groningen and University Medical Center Groningen, Groningen, Netherlands; ^3^Department of Orthodontics, University of Groningen and University Medical Center Groningen, Groningen, Netherlands

**Keywords:** bacterial biofilm, cascade reaction, infection, glucose, hydrogen peroxide, ROS

## Abstract

Cascade reactions integrate two or more reactions, of which each subsequent reaction can only start when the previous reaction step is completed. Employing natural substrates in the human body such as glucose and oxygen, cascade reactions can generate reactive oxygen species (ROS) to kill tumor cells, but cascade reactions may also have potential as a direly needed, novel bacterial infection-control strategy. ROS can disintegrate the EPS matrix of infectious biofilm, disrupt bacterial cell membranes, and damage intra-cellular DNA. Application of cascade reactions producing ROS as a new infection-control strategy is still in its infancy. The main advantages for infection-control cascade reactions include the fact that they are non-antibiotic based and induction of ROS resistance is unlikely. However, the amount of ROS generated is generally low and antimicrobial efficacies reported are still far <3–4 log units necessary for clinical efficacy. Increasing the amounts of ROS generated by adding more substrate bears the risk of collateral damage to tissue surrounding an infection site. Collateral tissue damage upon increasing substrate concentrations may be prevented by locally increasing substrate concentrations, for instance, using smart nanocarriers. Smart, pH-responsive nanocarriers can self-target and accumulate in infectious biofilms from the blood circulation to confine ROS production inside the biofilm to yield long-term presence of ROS, despite the short lifetime (nanoseconds) of individual ROS molecules. Increasing bacterial killing efficacies using cascade reaction components containing nanocarriers constitutes a first, major challenge in the development of infection-control cascade reactions. Nevertheless, their use in combination with clinical antibiotic treatment may already yield synergistic effects, but this remains to be established for cascade reactions. Furthermore, specific patient groups possessing elevated levels of endogenous substrate (for instance, diabetic or cancer patients) may benefit from the use of cascade reaction components containing nanocarriers.

## Introduction

Bacterial infections have threatened mankind ever since its first existence. Infection control gained a major success with the discovery of antibiotics in 1923. However, this success lasted less than a century, and toward the turn of the century, the first reports of antibiotic-resistant bacterial pathogens appeared (Davies and Davies, 2010). The latest new antibiotic class was discovered in 1986, after which shortening of the effective lifetime of new antibiotics, and financial and regulatory hurdles cooled down the passion of pharmaceutical companies to develop new antibiotics. It is estimated that the number of deaths attributable to antimicrobial-resistant bacterial infections will rise to 10 million per year by 2050, at a cost of more than US$100 trillion (O'Neill, 2014).

An additional problem in infection control, next to antibiotic resistance, is the presentation of infectious bacteria in a biofilm mode of growth. In a biofilm mode of growth, adhering bacteria produce a matrix of extracellular polymeric substances (EPS), in which they protect themselves against the host immune system and environmental attacks, such as posed by UV exposure, pH changes, and antibiotics (Hall-Stoodley et al., 2004). The EPS matrix impedes penetration of antibiotics, yielding survival of bacteria residing in the depth of a biofilm, which necessitates long-term and high-dose antimicrobial treatment to eradicate infectious biofilms, not seldom followed by recurrence of the infection after treatment (Fux et al., 2005).

To counter the increasing threat of bacterial infections, numerous nanotechnology-based antimicrobials and smart, antimicrobial-delivery nanocarriers are being designed (Liu et al., 2019a), such as carbon quantum dots, graphene, gold, silver, iron oxide, or polymeric nanoparticles, including micelles or antimicrobial dendrimers. However, the antimicrobial efficacy of many new nanoparticles are insignificant for clinical usage and only achieve about 90% reductions in bacterial viability (1 log unit), while a minimum of 99.9–99.99% (3–4 log units) is required to achieve any clinical efficacy (Liu X. et al., 2019). ROS are sometimes called “the sword of nanotechnology,” and taking lessons from cancer therapy, several methods have become available to generate ROS (Sun et al., 2014; Liu X. et al., 2019). ROS produced by different types of nanoparticles in combination with the addition of external H_2_O_2_ (Gao et al., 2016; Liu et al., 2018; Naha et al., 2019) has been shown to be effective in biofilm eradication. However, the use of cascade reactions to counter bacterial infections through ROS production using endogenously present substrate, i.e., without addition of external H_2_O_2_, has not yet been extensively considered.

**Figure 1:**
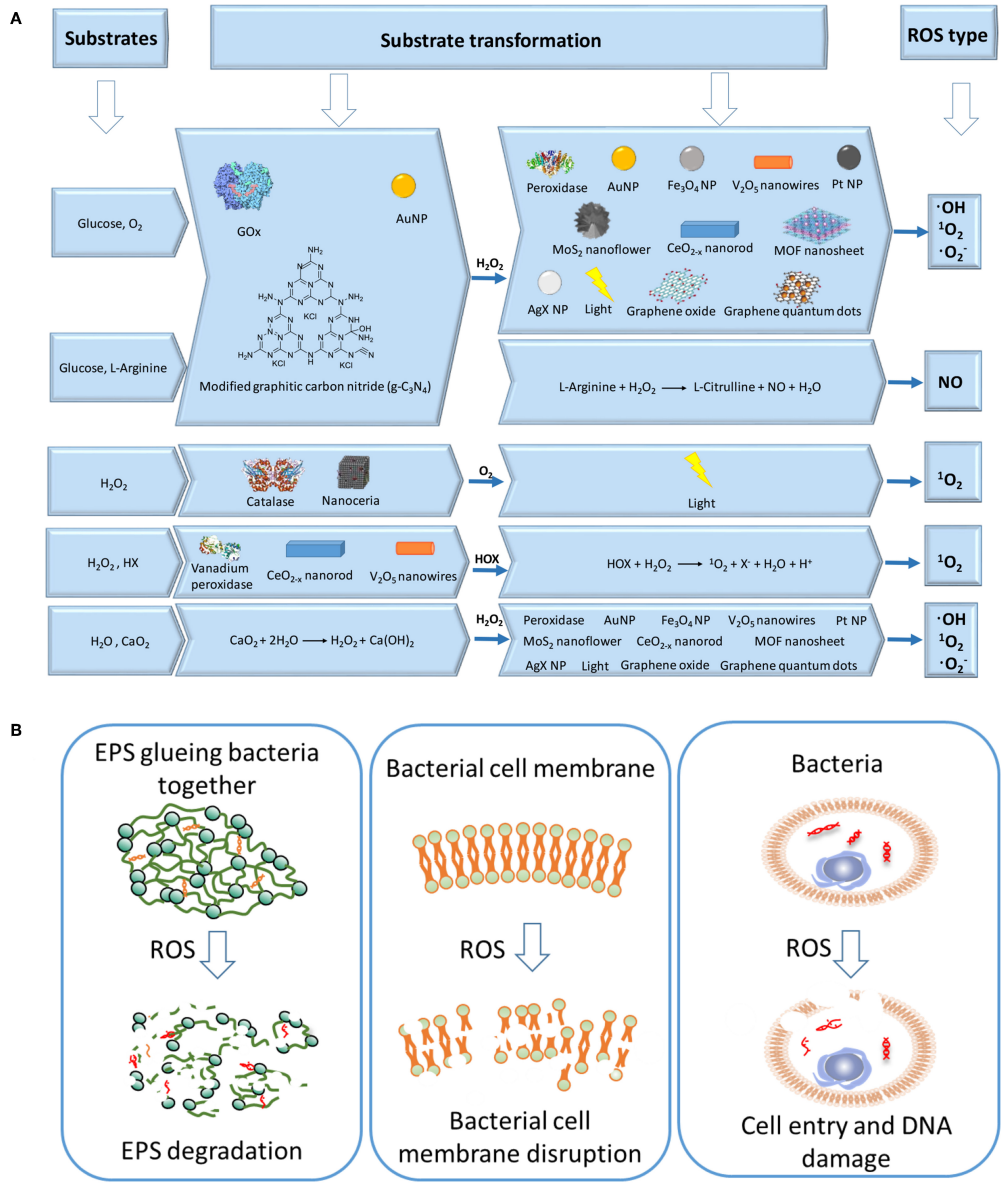
Schematics of cascade reactions for combating infectious biofilms. **(A)** Different substrates and enzymes involved in substrate transformations to generate different types of ROS. **(B)** ROS can combat infectious biofilms by degrading the EPS matrix of an infectious biofilm (Yan et al., 2018), disrupting bacterial cell membranes (Wang et al., 2017), and damaging intra-cellular DNA of biofilm inhabitants (Rowe et al., 2008; Cadet and Wagner, 2013).

A cascade reaction, also called tandem or domino reaction, is a chemical process that integrates at least two reactions, of which each subsequent reaction can only start when the previous reaction step is completed (Ricca et al., 2011). Enzymatic cascade reactions combine a series of enzymatic substrate transformations to produce a final product. In liner cascade reactions, the product of the first enzymatic reaction becomes the substrate of a second reaction (Ricca et al., 2011). Enzymatic cascade reactions widely occur in living organisms (Ricca et al., 2011). Photosynthesis and aerobic respiration, for instance, are enzymatic cascade reactions producing carbohydrates and carbon dioxide, respectively. Industrially, enzymatic cascade reactions are used in the one-pot synthesis of chemicals. Liner cascade reactions are the most widely used type of cascade reaction in cancer and infection therapy. Using naturally occurring substrates in the human body such as endogenous glucose and oxygen, cascade reactions can be used to produce highly toxic ROS (Figure 1A) (Yan et al., 2018), as an anti-cancer drug (Li et al., 2017) or antimicrobial (André et al., 2011; Liu et al., 2017; Liu X. et al., 2019) (Figure 1B).

In this review, we will focus on recent progress in the design of cascade reactions as a new strategy in infection control, taking lessons from current developments aimed toward using cascade reactions in cancer therapy. Use of cascade reactions for infection control is slowly emerging, yet offering equal or more perspective than new antibiotics or non-ROS-based infection-control strategies, because hitherto bacterial resistance to ROS has not been reported and is generally considered highly unlikely to develop.

## Chemistry of Cascade Reactions

In this section, different kinds of cascade reactions are classified by substrate. The conditions and mechanisms to achieve these cascade reactions are summarized.

### Glucose and Oxygen

Glucose is the most important carbohydrate in the human body and can yield gluconic acid and H_2_O_2_ upon reaction with oxygen (Itskov and Carlos, 2013; Fan et al., 2017), according to

(1) $$\begin{array}{l} \text{Glucose}+{\text{O}}_{2}\to \text{Gluconicacid}+{\text{H}}_{2}{\text{O}}_{2} \end{array}$$

**Figure 2:**
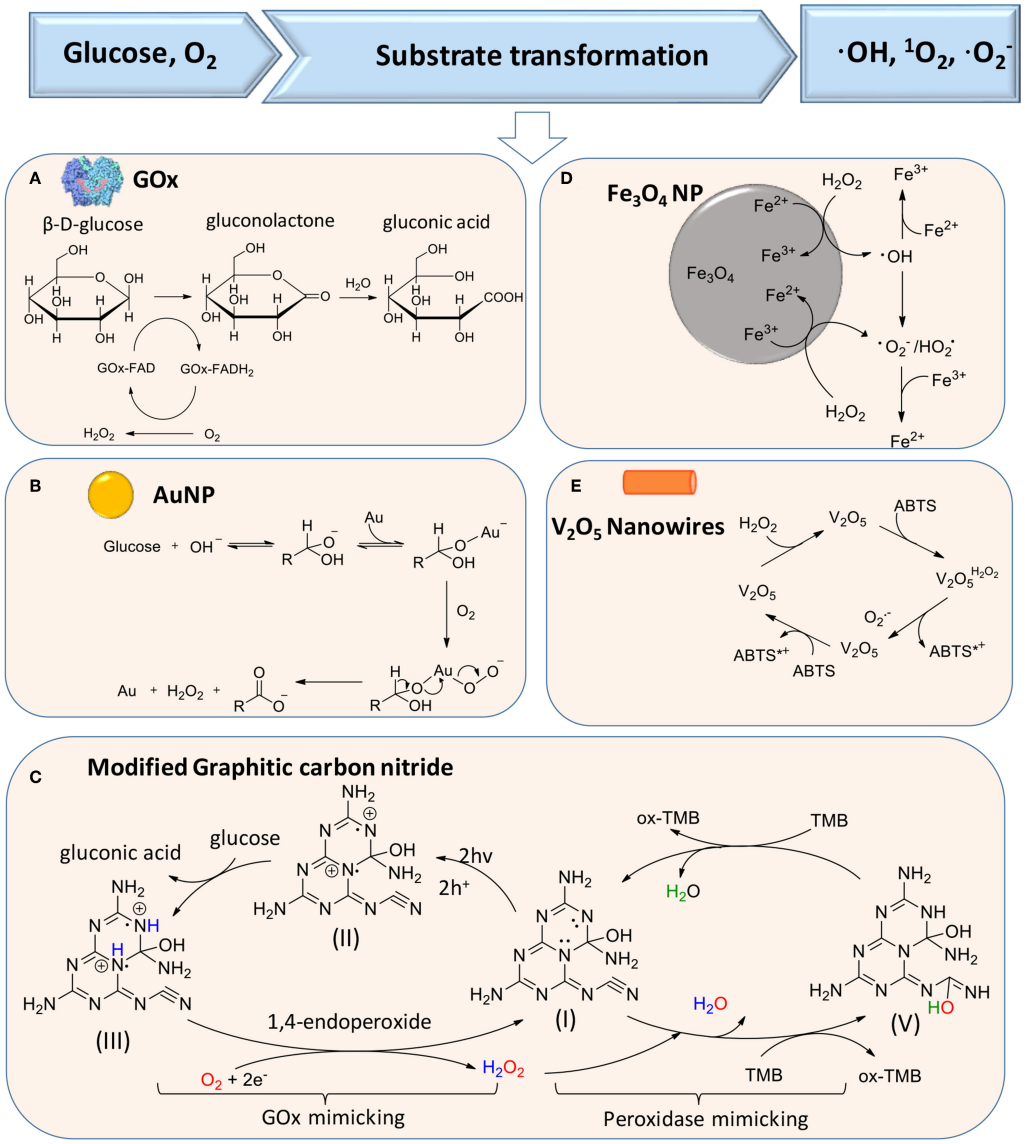
Substrate transformations in cascade reactions generating ROS, based on glucose and oxygen as substrates. **(A)** Generation of H_2_O_2_ from glucose using GOx (Witt et al., 2000) (with permission of Portland Press). **(B)** Catalysis of glucose transformation to H_2_O_2_ using catalytic, gold nanoparticles (Comotti et al., 2006) (with permission of John Wiley and Sons). **(C)** Modified carbon nitride as a bifunctional glucose-peroxidase enzyme-mimic (Zhang et al., 2019) (with permission of Nature Publishing Group). **(D)** Activation of H_2_O_2_ on Fe_3_O_4_ nanoparticles to achieve transformation of H_2_O_2_ to ROS (Wang et al., 2010) (with permission of Elsevier). **(E)** V_2_O_5_ nanowires catalyze transformation of H_2_O_2_ to ${\cdot \text{O}}_{2}^{-}$ in the presence of ABTS [2,2′-azinobis(3-ethylbenzthiazoline-6-sulfonate)] (André et al., 2011) (with permission of John Wiley and Sons).

The production of H_2_O_2_ according to reaction (1) can be catalyzed by glucose oxidase (GOx), as the first reaction in a liner cascade reaction (Figure 1A). This catalytic process can be divided into a reductive and oxidative half-reaction, as shown in Figure 2A. In the reductive half-reaction, β-D-glucose loses two electrons to form δ-gluconolactone, which is subsequently hydrolyzed to gluconic acid. After receiving two electrons from the reductive half-reaction, the flavin ring of GOx becomes reduced to FADH_2_ after which, in the oxidative half-reaction, the same two electrons transferred from GOx-FADH_2_ to oxygen yield H_2_O_2_ and GOx in an oxidized state (Witt et al., 2000). Reaction (1) can also be catalyzed by gold nanoparticles and modified graphitic carbon nitride, as shown schematically in Figures 2B,C, respectively.

In the second reaction of the cascade, different artificial, catalytic nanoparticles can be employed to transform H_2_O_2_ into ·OH, ^1^O_2_ (singlet oxygen), and ${\cdot \text{O}}_{2}^{-}$ (Cho et al., 2019). These catalytic nanoparticles include AuNPs (Zhang et al., 2019), iron oxide (Fan et al., 2012; Duan et al., 2015; Gao et al., 2016; Liu et al., 2018; Naha et al., 2019), silver halides (Wang et al., 2014), platinum (Liu X. et al., 2016; Wu et al., 2018), cerium oxide (Niu et al., 2007; Celardo et al., 2011), vanadium oxide (André et al., 2011), 2D metallo-porphyrinic metal-organic framework (MOF) nanosheets (Huang et al., 2017), and molybdenum disulfide (MoS_2_) nanoflowers (Yin et al., 2016). Metal-free artificial catalysts include modified carbon nitride (Yin et al., 2016), graphene oxide (Song et al., 2010), and graphene quantum dots (Sun et al., 2014; Duan et al., 2015). Importantly, these catalytic nanoparticles can be functionally modified to enhance their catalytic activity. However, mechanisms to transform H_2_O_2_ into ·OH, ^1^O_2_, and ${\cdot \text{O}}_{2}^{-}$ are different for different artificial catalytic nanoparticles. Proposed catalytic mechanisms for iron oxide nanoparticles and V_2_O_5_ nanowires are summarized in Figures 2D,E (Natalio et al., 2012). Light irradiation can also be used as a catalytic mediator to split H_2_O_2_ into two ·OH (Chang et al., 2017).

### Glucose and L-arginine

**Figure 3:**
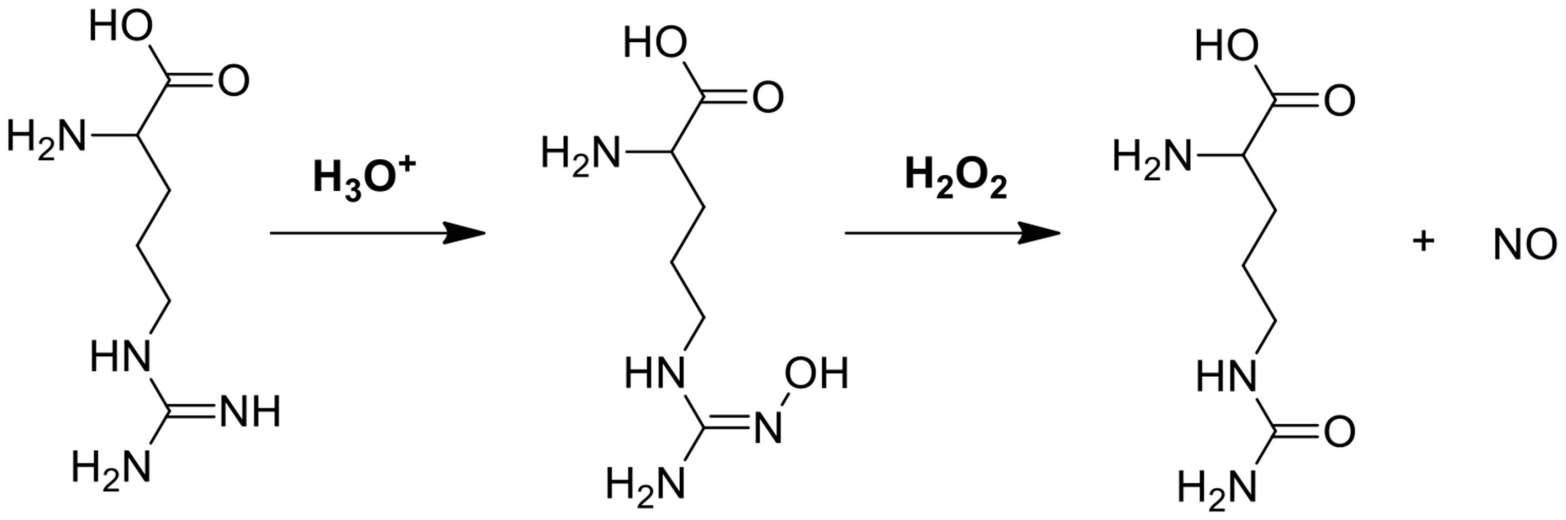
The mechanism of NO generation through reaction between L-arginine and H_2_O_2_ (Fan et al., 2017).

In another cascade reaction involving glucose (Figure 1A), L-arginine can be employed to produce highly toxic nitric oxide (NO) by oxidation of the guanidine function of L-arginine (see also Figure 3). Interestingly, gluconic acid, as a by-product in reaction (1), leads to a pH decrease, accelerating L-arginine catalytic transformation of H_2_O_2_ into NO (Fan et al., 2017).

### H_2_O_2_

**Figure 4:**
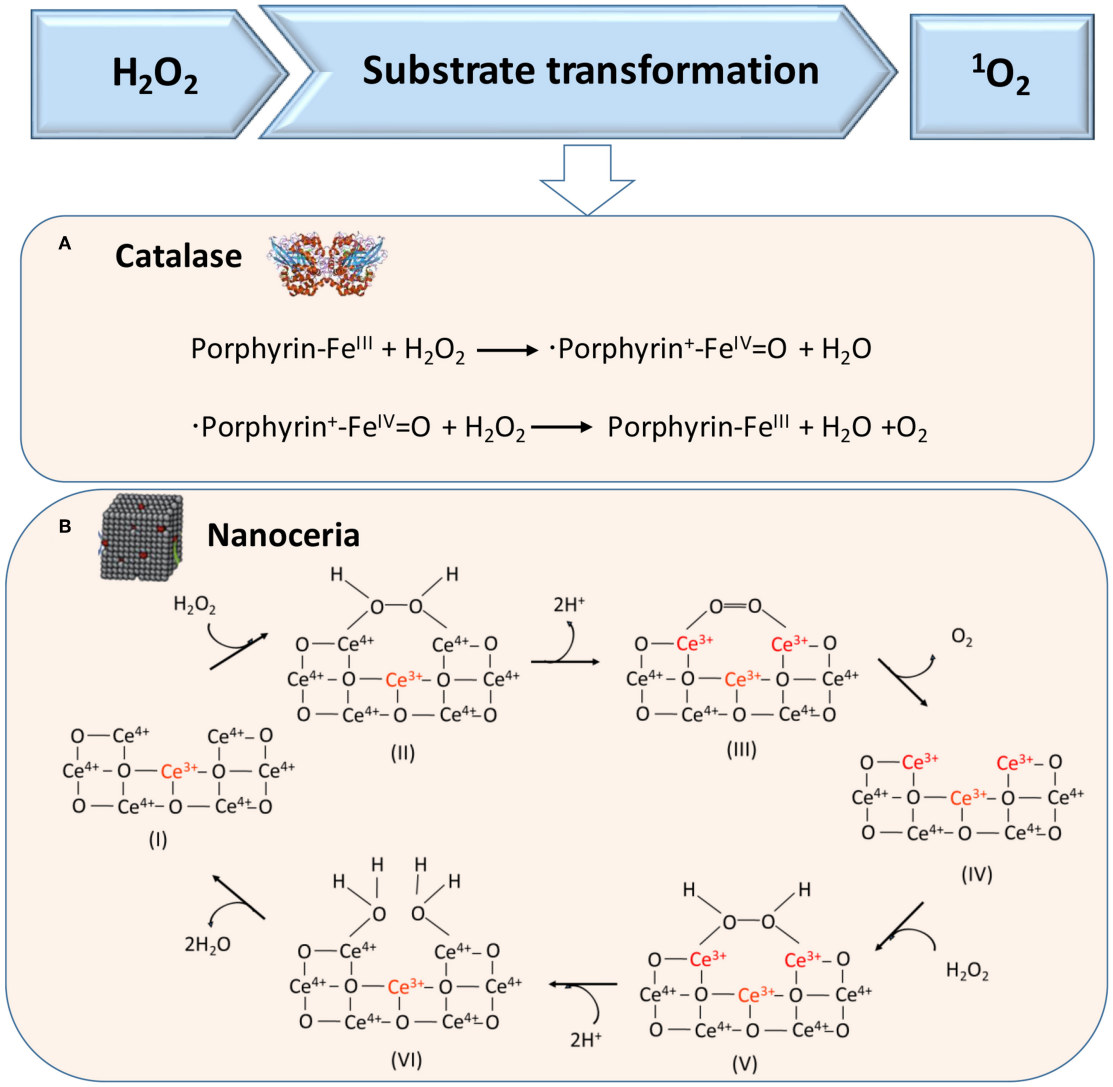
Proposed mechanisms of enzymatic transformations using H_2_O_2_ as a substrate. **(A)** Transformation of H_2_O_2_ by catalase into O_2_ (Alfonso-Prieto et al., 2009). **(B)** Transformation of H_2_O_2_ using catalytic cerium oxide nanoparticles (“nanoceria”) into O_2_ (Celardo et al., 2011) (with permission of the Royal Society of Chemistry).

H_2_O_2_ can also act as a substrate for generating ^1^O_2_ in cascade reactions, as catalyzed in the first cascade reaction by catalase, a tetrameric heme protein (Figure 4A) (Alfonso-Prieto et al., 2009) or artificial, catalytic nanoparticles, such as cerium oxide nanoparticles (Figure 4B) (Herget et al., 2017). In the second reaction, also light can be used to transform O_2_ into singlet oxygen, which requires the presence of a suitable photosensitizer (Li et al., 2017), such as tetrapyrroles (absorption band around 400 nm), porphyrins (absorption band around 630 nm), chlorins (absorption band around 650–690 nm), or “bacteriochlorins” with an absorption band shifted further into the red (Castano et al., 2004).

### H_2_O_2_ and halides

**Figure 5:**
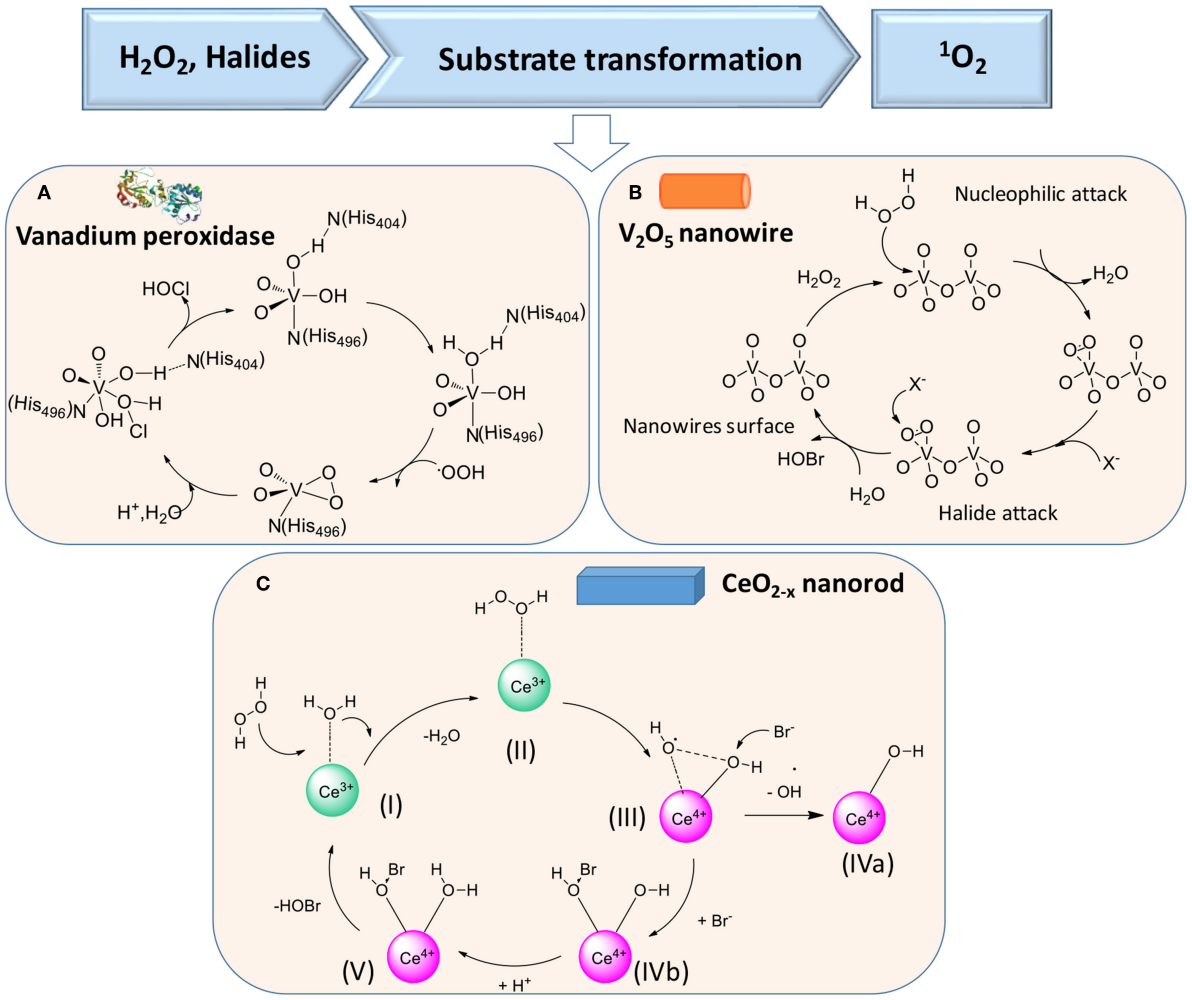
Proposed mechanisms of enzymatic transformations using H_2_O_2_ and halides as substrates. **(A)** Transformation of H_2_O_2_ and halides by vanadium peroxidase (Ligtenbarg et al., 2003) (with permission of Elsevier). **(B)** Transformation of H_2_O_2_ and halides by V_2_O_5_ nanowires (Natalio et al., 2012) (with permission of Nature Publishing Group). **(C)** CeO_2−x_ nanorod as catalase (Herget et al., 2017) (with permission of John Wiley and Sons).

In the presence of H_2_O_2_ and halides X^−^ [such as chloride (Cl^−^) or bromide (Br^−^) ions], vanadium peroxidase (see Figure 5A), V_2_O_5_ nanowires (Natalio et al., 2012) (Figure 5B), and CeO_2−x_ nanorods (Herget et al., 2017) (Figure 5C) can also be used in the first cascade reaction [see reaction (2)], followed by the second reaction [reaction (3)]

(2) $$\begin{array}{l} {\text{H}}_{2}{\text{O}}_{2}+{\text{X}}^{-}+{\text{H}}^{+}\to {\text{H}}_{2}\text{O}+\text{HOX} \end{array}$$

(3) $$\begin{array}{l} \text{HOX}+{\text{H}}_{2}{\text{O}}_{2}\to^{1}{\text{O}}_{2}+{\text{X}}^{-}+{\text{H}}_{2}\text{O}+{\text{H}}^{+} \end{array}$$

### H_2_O and CaO_2_

An H_2_O_2_-free cascade reaction is based on water (H_2_O) and calcium peroxide (CaO_2_), as substrates (Figure 1A) according to Yan et al. (2018)

(4) $$\begin{array}{l} \text{Ca}{\text{O}}_{2}+2{\text{H}}_{2}\text{O}\to {\text{H}}_{2}{\text{O}}_{2}+\text{Ca}{\left(\text{OH}\right)}_{2} \end{array}$$

CaO_2_ reacts with H_2_O through a redox reaction, in which CaO_2_ as a reducing agent loses four electrons. After obtaining the corresponding electrons, H_2_O is oxidized to H_2_O_2_ after which, in the second reaction of the cascade, H_2_O_2_ can be transformed into ·OH, ^1^O_2_ and ${\cdot \text{O}}_{2}^{-}$ by any of the reactions described in sections Glucose and Oxygen and Glucose and L-Arginine.

## Application of Cascade Reactions in Cancer Therapy

**Figure 6:**
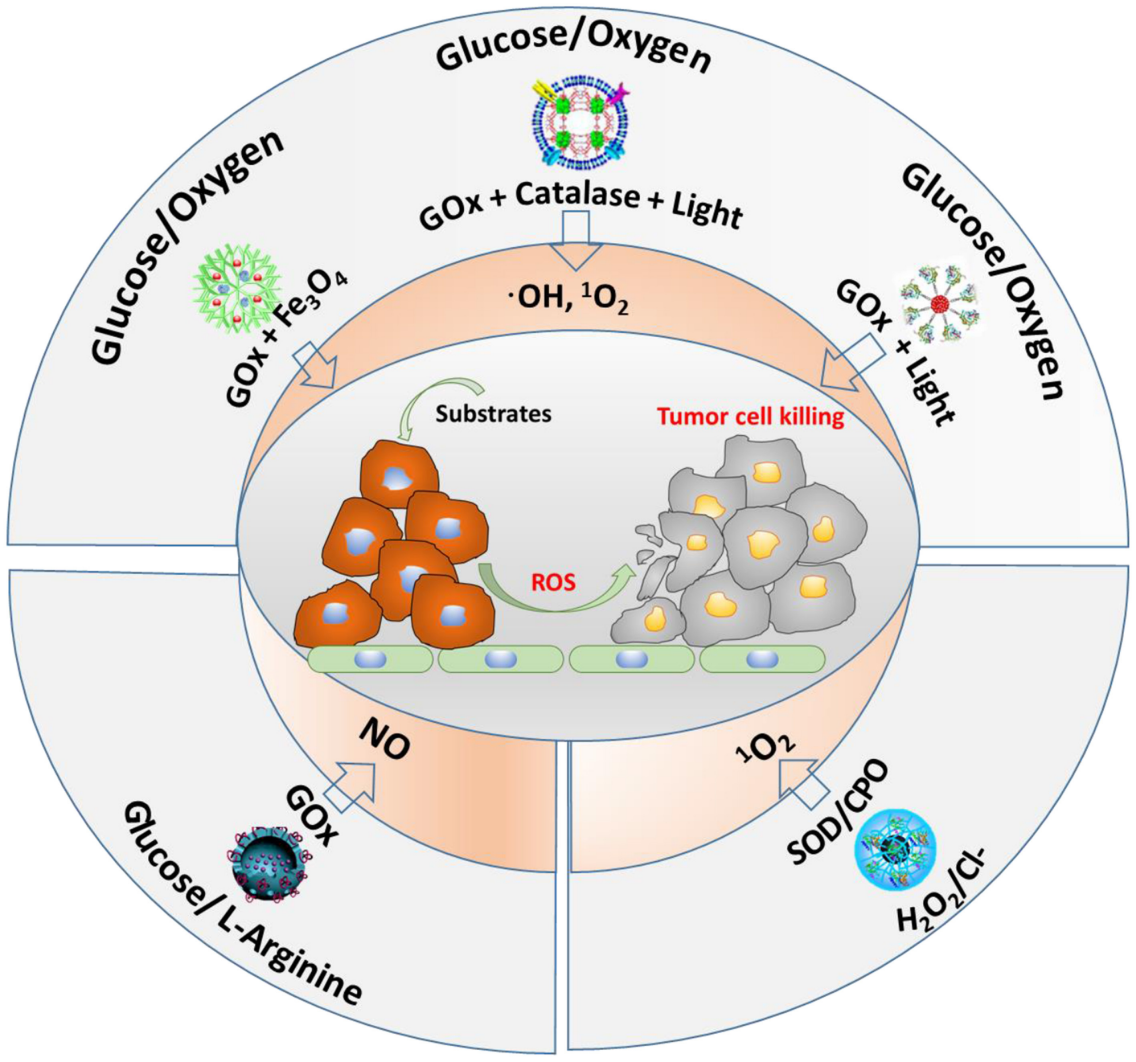
Different cascade reactions considered for tumor treatment, using glucose, O_2_, H_2_O_2_, and Cl^−^ (endogenously available) and L-arginine (not endogenously available) as substrates, yielding different types of ROS.

The main substrates and enzymes in cascade reactions producing ROS that are currently considered for anti-tumor therapy involve glucose and oxygen, glucose and L-arginine, or H_2_O_2_/Cl^−^ as substrates (Figure 6). GOx and Fe_3_O_4_ nanoparticles have been contained into dendritic silica nanocarriers to initiate a cascade reaction, using endogenous glucose present in tumor cells to generate H_2_O_2_ (Huo et al., 2017). As a second step, the Fe_3_O_4_ nanoparticles catalyze H_2_O_2_ into highly toxic ·OH. *In vivo*, this cascade reaction demonstrated suppression of mammary tumor growth. Instead of using GOx and Fe_3_O_4_ nanoparticles in dendritic silica to produce ROS (hydroxyl radicals), GOx and catalase have also been integrated in MOF nanocarriers (Li et al., 2017). Here, as the second step in the cascade reaction, H_2_O_2_ produced endogenously or from oxidation of glucose by GOx, is converted to oxygen by catalase. After that, singlet oxygen (^1^O_2_) is generated under 660-nm light irradiation (Li et al., 2017). Alternatively, another cascade reaction involving photodynamics based on one enzyme was described (Chang et al., 2017), in which GOx was conjugated to polymer dots to convert glucose into H_2_O_2_. Under light irradiation, the H_2_O_2_ generated was photolyzed to hydroxyl radicals.

Glucose and L-arginine have also been used as substrates in cascade reactions to produce NO (see also Figure 6) in order to kill tumor cells (Fan et al., 2017). To this end, GOx and L-arginine have been contained in hollow, mesoporous organosilica nanocarriers, and after generation of H_2_O_2_ from glucose present in tumor sites using GOx, reaction of H_2_O_2_ with L-arginine generated NO, which was shown to prolong the life of tumor-bearing mice.

Finally, endogenous ^**·**^${\text{O}}_{2}^{-}$ and Cl^−^ have been used as substrates in cascade reactions (Wang et al., 2014). Using superoxide dismutase (SOD) and chloroperoxidase (CPO), endogenous ^**·**^${\text{O}}_{2}^{-}$ can be catalyzed by SOD to H_2_O_2_, which is subsequently further oxidized with the aid of Cl^−^ and CPO to ^1^O_2_. This cascade reaction showed negligible toxicity to healthy cells, but was highly toxic to tumor cells.

## Application of Cascade Reactions for Bacterial Infection Control: Lessons From Tumor Treatment

Based on the similarity between tumor sites and bacterial biofilms (Table 1), we can take lessons from the cascade reactions considered for tumor therapy, as shown in Figure 6. All endogenous substrates used for cancer therapy are available in bacterial infection sites, and ROS shows highly toxic toward all bacterial pathogens, regardless of their Gram character (Vatansever et al., 2013). Therefore, cascade reactions are nowadays started to be considered as an alternative strategy for the control of infectious biofilms. The number of studies applying cascade reaction for bacterial infection control is limited however (see overview in Table 2), but their results warrant analysis and offer future perspective that are new for infection control.

**Table 1:** Similarities between tumor sites and infectious biofilms, stimulating the application of cascade reactions in infectious bacterial biofilms, taking lessons from their application in tumor treatment.

**Similarity**	**Tumor site**	**Infectious biofilm**
Hampered transport	Tumors larger than 2 mm^3^, limit oxygen diffusion (Trédan et al., 2007; Danhier et al., 2010).	The EPS matrix limits transport of nutrients to the depth of a biofilm (Flemming et al., 2007; Billings et al., 2015).
Acidic pH	Acidic pH between 6.0 and 7.0 (Justus et al., 2013).	Acidic pH < 6.0 (Koo et al., 2017; Liu et al., 2019a).
Endogenous substrate availability	High killing efficacy of ROS toward tumor cells (Postiglione et al., 2011).	ROS can destruct the EPS matrix and kill biofilm bacteria (Walch et al., 2015).
Clinical treatment	Occurrence of resistance against chemotherapeutics, recurrence of tumor growth (Mansoori et al., 2017).	Occurrence of resistance against antimicrobials, recurrence of infection (Aslam et al., 2018).

**Table 2:** Summary of cascade reactions considered for infection control, including substrates required, nanocarriers of cascade reaction components, types of ROS generated, and antimicrobial activities observed.

**Substrate**	**Nanocarrier**	**ROS**	**Antimicrobial activity**	**References**
Glucose/Oxygen	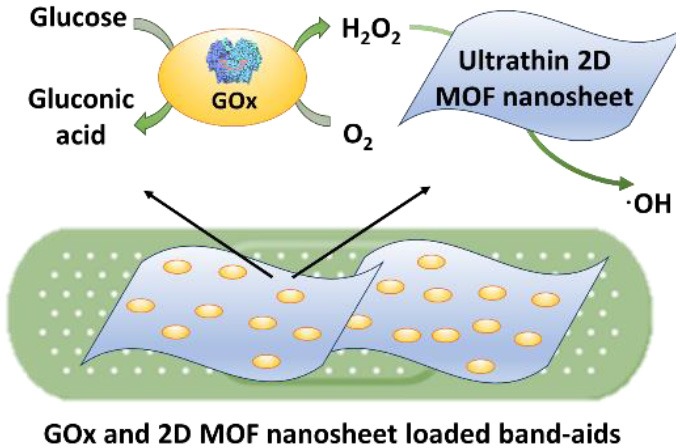	·OH	- Against planktonic *S. aureus* and *E. coli* - Improved infected wound healing in mice	Liu X. et al., 2019
	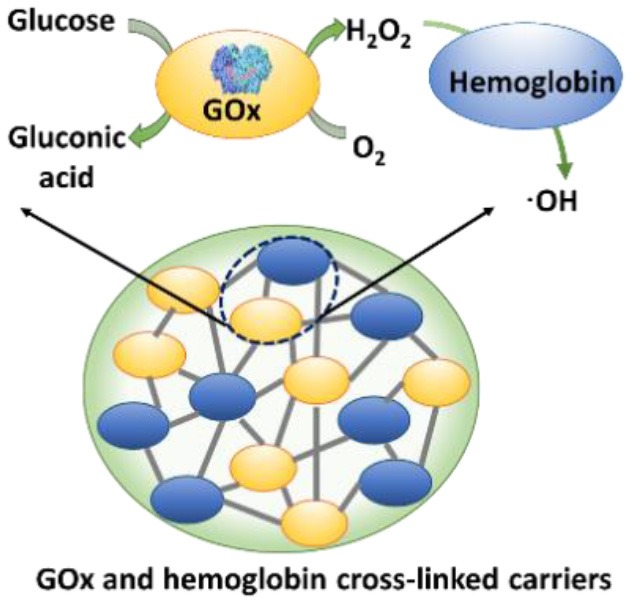	·OH	- Against planktonic MRSA - Inhibiting MRSA biofilm formation	Li et al., 2019
CaO_2_/H_2_O	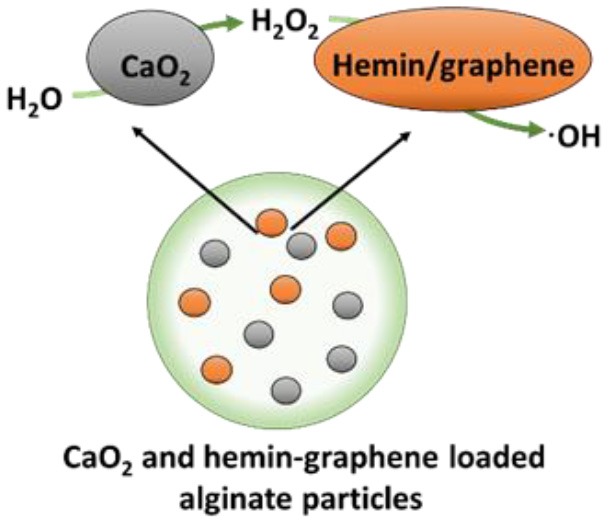	·OH	- Against planktonic *S. aureus* and *E. coli* - Inhibiting *S. aureus* biofilm formation - Dispersing *S. aureus* biofilm - *In vivo* implant-related periprosthetic infection of mice	Yan et al., 2018
H_2_O_2_/Br^−^	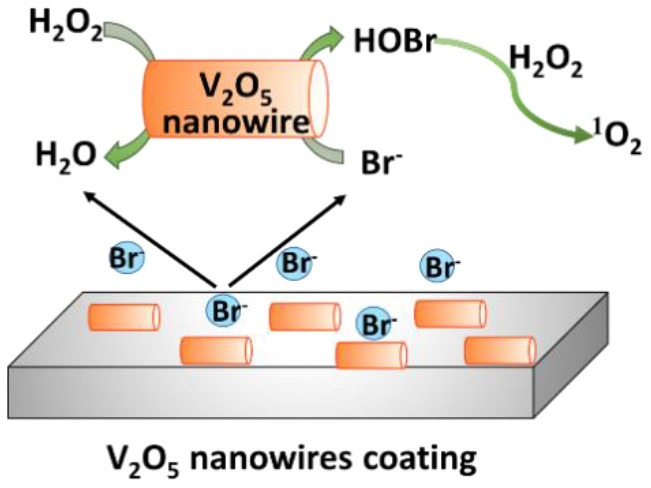	^1^O_2_	- Against planktonic *S. aureus* and *E. coli* - Inhibiting *S. aureus*, and *E. coli* biofilm formation	Natalio et al., 2012

### Cascade Reactions Considered for Bacterial Infection Control

Application of cascade reactions as a new infection-control strategy is currently in its infancy, and to our knowledge, only four studies so far have considered the use of cascade reactions for infection control (see Table 2) that employ different nanocarriers for the cascade reaction components.

**Figure 7:**
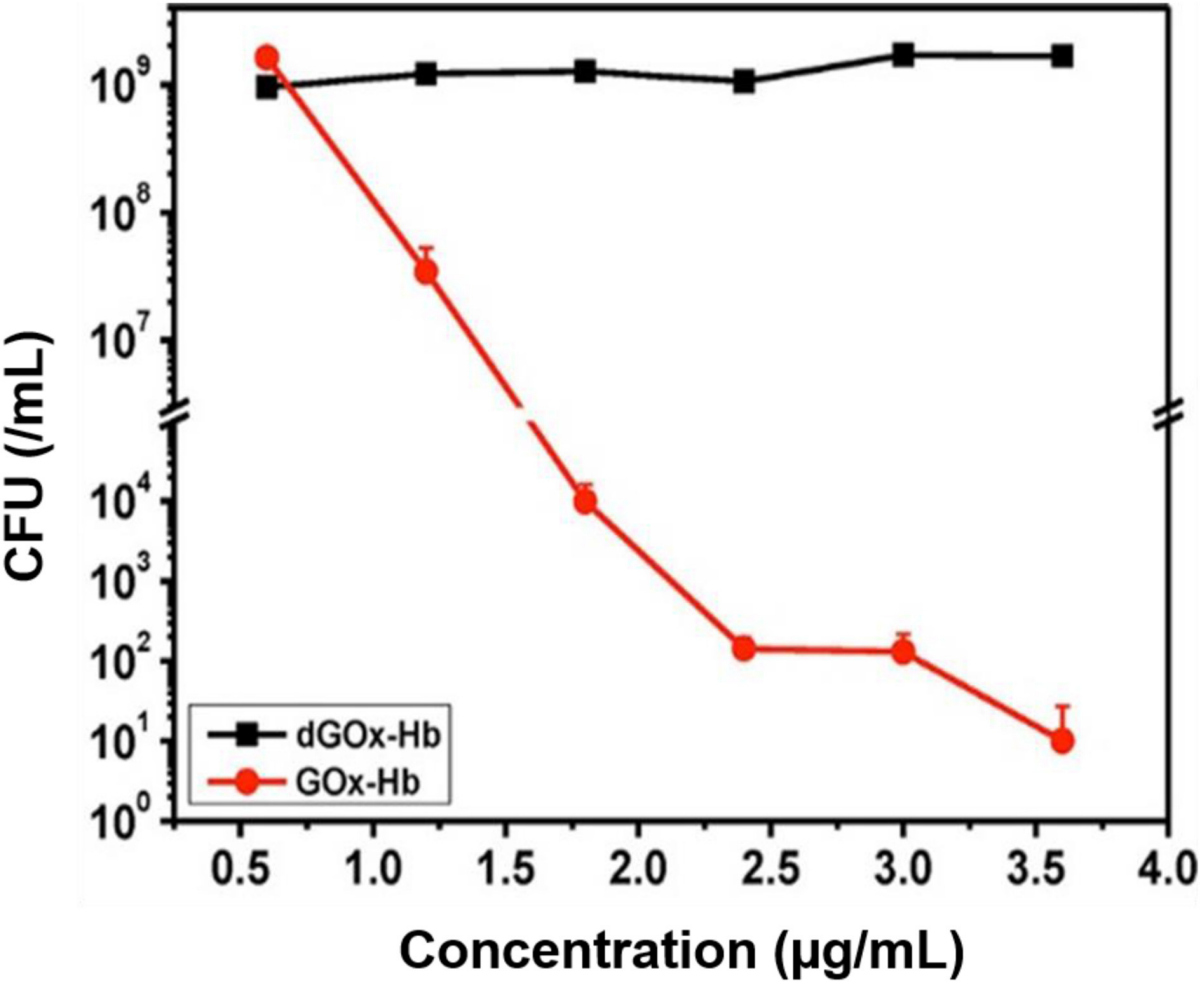
CFUs (MRSA) in growth medium supplemented with glucose (12.5 mM) after 24 h of growth, as a function of GOx-Hb nanocarrier concentration. For control, nanocarriers with denatured GOx (dGOx-Hb) were included (Li et al., 2019) (with permission of ACS).

Two cascade reactions based on glucose and O_2_ as substrate have been evaluated for their antimicrobial activity. GOx absorbed in ultrathin two-dimensional (2D) MOF nanosheet carriers (Liu X. et al., 2019), *in vitro* generated ROS that killed planktonic *Escherichia coli* and *Staphylococcus aureus* after 5 h incubation with 15 mM glucose in the growth medium. Bacterial killing was far lower (88 and 90% for *E. coli* and *S. aureus*, respectively) than the 99.9–99.99% efficacy limit considered to be required for clinical efficacy (Liu et al., 2019a). However, when locally applied as a 2D MOF/GOx band-aid on infected (3 × 10^7^ CFU/site *S. aureus*) wounds in mice, mice treated with 2D MOF/GOx band-aids containing 50 μl of a 10 mM glucose solution showed faster wound healing after 3 days of treatment, retrieving less (91% reduction) CFUs than when treated with blank band-aids. Glucose and O_2_ were also used as substrates in combination with another peroxidase, hemoglobin (Hb) to catalyze H_2_O_2_ into ^·^OH. Cascade reaction components were contained in MnCO_3_ nanocarriers (Li et al., 2019). In growth medium supplemented with glucose to a concentration of 12.5 mM, i.e., substantially higher than endogenously occurring (before eating <6.1 mM; Stumvoll et al., 2005), GOx-Hb nanocarriers yielded 7 log unit inhibition in planktonic MRSA growth (Figure 7). In addition, biomass analysis based on Crystal Violet staining indicated the ability of GOx-Hb nanocarriers to inhibit MRSA biofilm formation during 48 h exposure to GOx-Hb and incubation in glucose supplemented growth medium. Nanocarriers containing denatured GOx did not inhibit biofilm formation.

A cascade reaction using CaO_2_ and H_2_O as substrates to generate hydroxyl radicals was fabricated by containing CaO_2_ and hemin-carrying graphene into alginate (Yan et al., 2018). Importantly, these alginate nanocarriers did not contain any H_2_O_2_ as a substrate and CaO_2_ and H_2_O were locally converted into ROS. Planktonically, these nanocarriers yielded relatively low killing of *S. aureus* and *E. coli* (90 and 95%, respectively, after 6 h exposure). Also, 72-h *S. aureus* biofilm growth in the presence of the cascade reaction components containing nanocarriers was low, yielding <5-μm-thick biofilms vs. 25 μm in their absence. In addition, exposure of an existing *S. aureus* biofilm to the cascade reaction components containing nanocarriers eradicated 81% of its inhabitants. Interestingly, these cascade reaction components containing nanocarriers caused biofilm dispersal, degrading DNA and proteins, as major components of the EPS-matrix holding a biofilm together. Finally, *S. aureus*-infected wounds (1 × 10^5^ CFU/site) in rats gradually healed after 7 days (>90% bacterial killing) when treated with these alginate nanocarriers, with obvious swelling and pus formation in control groups.

H_2_O_2_ has also been used in combination with Br^−^ as substrates in the presence of V_2_O_5_ nanowires, with an intended, initial use in the marine environment (Natalio et al., 2012). V_2_O_5_ nanowires can work as natural haloperoxidases to transform bromide ions to hypobromous acid (HOBr) in the presence of H_2_O_2_ (Natalio et al., 2012). The antimicrobial activity against human pathogens was low, however, decreasing planktonic growth of *E. coli* by only 78% and of *S. aureus* by only 96% in the presence of V_2_O_5_ nanowires (0.075 mg/ml), Br_2_ (1 mM), and H_2_O_2_ (10 μM), and as compared to bacteria grown in the absence of additives.

### Advantages and Disadvantages of Cascade Reactions for Biofilm Control

The main advantages for infection-control cascade reactions include the fact that they are non-antibiotic based. Bacteria possess little or no resistance against ROS, although some bacterial species can develop resistance against specific ROS species, such as ^**·**^${\text{O}}_{2}^{-}$ and H_2_O_2_, but not against ^**·**^OH and ^1^O_2_ (Vatansever et al., 2013). Often, however, different species of ROS are generated at the same time in catalytic reactions. ROS as generated in cascade reactions kills both Gram-positive and Gram-negative bacterial strains in a non-specific way. On the one hand, this may be considered an advantage, but on the other hand, the commensal microflora can also be affected, unless the cascade reaction can be localized in or near the infection site. Although nanocarriers and artificial enzymes used in cascade reactions are relatively easy to synthesize and modify, confining ROS generation to an infection site may be more troublesome.

As a drawback of the current status of cascade reactions, the amount of ROS generated is generally low and antimicrobial efficacies reported are below the limit of 3–4 log units considered necessary for clinical efficacy (Yan et al., 2018; Liu et al., 2019a). Although the amount of ROS generated can be enhanced by adding more substrate (Fan et al., 2017), this needs to be done carefully in order to prevent collateral damage to tissue surrounding an infection site. This, too, requires confining of ROS generation to an infection site.

## Perspectives of the Use of Cascade Reactions for Infection Control

The antimicrobial activity of ROS (Rowe et al., 2008; Cadet and Wagner, 2013; Vatansever et al., 2013; Gao et al., 2016; Wang et al., 2017; Liu et al., 2018; Yan et al., 2018) and the potential advantages of the use of cascade reaction as a new infection-control strategy warrant their further development for infection control, but several challenges have to be overcome before their clinical use becomes into sight. The biggest challenge in the further development of infection-control cascade reactions is to increase their bacterial killing efficacy to levels that can be expected to be clinically effective (i.e., minimally 3–log unit reductions in CFUs, equal to minimal percentage reductions of 99.9–99.99%). This should preferentially be done using endogenously available substrates, but this option should be carefully balanced against the potential damage that high concentrations of ROS can do to tissue cells surrounding an infection site (Li et al., 2019). Alternatively, additional substrate can be locally administered to increase local ROS generation, which may reduce collateral tissue damage. Collateral tissue cell damage might be fully prevented by using self-targeting, pH-responsive nanocarriers that penetrate and accumulate in infectious biofilms (Liu Y. et al., 2016), to confine the occurrence of the cascade reaction and generation of ROS to inside the biofilm itself. A further advantage of such confinement would be that it will create long-term presence of ROS in a biofilm to enhance bacterial killing, despite the short lifetime (nanoseconds) of individual ROS molecules, arguably too short to yield effective bacterial killing.

Nevertheless, cascade reactions generating relatively low levels of ROS might still be clinically useful in combination with clinically applied antibiotics to enhance their fading efficacies (Nguyen et al., 2016). Synergistic action between different antimicrobials is a common phenomenon, and it is known to even yield killing of bacterial pathogens that are resistant to either of the antimicrobials when applied in monotherapy (Klahn and Brönstrup, 2017). This approach bears advantages for downward clinical translation of infection-control cascade reactions, because it builds on existing clinically applied antibiotics, from which further development of infection-control cascade reactions can be facilitated more easily. Moreover, it might increase the effective lifetime of current and new antibiotics to be developed (Liu et al., 2019b), with the potential to reduce the induction of antibiotic resistance (Mao et al., 2018).

As a final perspective of infection-control cascade reactions, specific patient groups with elevated endogenous substrate levels may benefit from cascade reactions. Diabetic patients, for instance, can suffer from difficult-to-heal infected wounds and have elevated glucose levels (ranging from ≥7.0 mM before to ≥11.1 mM after eating) compared with healthy glucose levels (ranging from <5.6 mM before to 7.8 mM after eating) (Stumvoll et al., 2005). Local generation of ROS through suitable cascade reactions may be extremely suitable for treating diabetic foot ulcers that have been demonstrated to be caused by hard-to-treat infectious biofilms (Neut et al., 2011). Cancer patients are another specific patient group for which infection-control cascade reactions could be useful. There is growing evidence that bacteria can metabolize chemotherapeutic drugs (Geller et al., 2017). Accordingly, intra-tumor bacteria may contribute to resistance against chemotherapeutic drugs among certain tumors. Since tumor sites possess elevated levels of H_2_O_2_ (50–100 μM around a tumor site vs. 20 nM elsewhere in the body) (De Garcia Lux et al., 2012), infection-control cascade reactions based on endogenous H_2_O_2_ substrate availability may play a role for this specific patient groups in treating tumor-associated infections.

In short, cascade reactions have potential as a new infection control strategy, but much work remains to be done to solve the challenges listed above in order to make cascade reactions into a real clinical addendum to the antimicrobial armamentarium of modern medicine.

## Author Contributions

All authors listed have made a substantial, direct and intellectual contribution to the work, and approved it for publication.

### Conflict of Interest

HB is also director of a consulting company, SASA BV. The remaining authors declare no conflicts of interest with respect to authorship and/or publication of this article. Opinions and assertions contained herein are those of the authors and are not construed as necessarily representing views of their respective employers.
